# Survival and self-renewing capacity of breast cancer initiating cells during fractionated radiation treatment

**DOI:** 10.1186/bcr2479

**Published:** 2010-02-16

**Authors:** Chann Lagadec, Erina Vlashi, Lorenza Della Donna, YongHong Meng, Carmen Dekmezian, Kwanghee Kim, Frank Pajonk

**Affiliations:** 1 Division of Molecular and Cellular Oncology, Department of Radiation Oncology, David Geffen School of Medicine at University of California, Los Angeles, 10833 Le Conte Avenue, Los Angeles, CA 90095-1714, USA; 2 Jonsson Comprehensive Cancer Center, University of California, Los Angeles, 8-684 Factor Building Box 951781, Los Angeles, CA 90095-1781, USA

## Abstract

**Introduction:**

Recent data indicate a hierarchical organization of many solid cancers, including breast cancer, with a small number of cancer initiating cells (CICs) that have the ability to self-renew and exhibit multi-lineage potency. We, and others, have demonstrated that CICs in breast cancer and glioma are relatively resistant to ionizing radiation if compared to their non-tumorigenic counterparts. However, the extent of the remaining self-renewing capacity of CICs after fractions of radiation is currently unknown. We hypothesized that CICs, in contrast to their non-tumorigenic counterparts, not only survive fractions of ionizing radiation but also retain the CIC phenotype as defined by operational means.

**Methods:**

We used two marker systems to identify breast CICs (CD24^-/low^/CD44^high^, or lack of proteasome activity) and performed sphere-forming assays after multiple clinical fractions of radiation. Lineage tracking was performed by membrane staining. Cell cycle distribution and RNA content were assessed by flow cytometry and senescence was assessed via β-galactosidase staining.

**Results:**

We demonstrated that irradiated CICs survived and retained their self-renewal capacity for at least four generations. We show that fractionated radiation not only spared CICs but also mobilized them from a quiescent/G0 phase of the cell cycle into actively cycling cells, while the surviving non-tumorigenic cells were driven into senescence.

**Conclusions:**

The breast CIC population retains increased self-renewal capacity over several generations and therefore, we conclude that increases in the number of CICs after sublethal doses of radiation have potential clinical importance. Prevention of this process may lead to improved clinical outcome.

## Introduction

Recent experimental data provide evidence for a hierarchical structure of many solid cancers [1-7] including breast cancer [8]. Using surface markers, a small population of highly tumorigenic breast cancer initiating cells (CICs) has been prospectively identified, which exhibits a cancer stem cell phenotype, thus being able to self-renew and to give rise to progeny of multiple different lineages of differentiated cells [8]. We previously demonstrated that this population of cells was relatively resistant against ionizing radiation if compared to their non-tumorigenic counterparts [9]. Two other groups have confirmed our data [10,11]. Additionally, Han and Crowe recently identified a side population in MCF7 and T47D breast cancer cells [12], which initiated tumors *in vivo *and was more resistant to ionizing radiation than the non-side population. Furthermore, we, and others, have reported that the population of CICs increases during the course of fractionated radiation [9,13]. However, the extent of the remaining self-renewing capacity of this CIC population after fractions of radiation is currently unknown. This information is crucial for evaluating the significance of radiation resistance of CICs.

Breast CICs were first prospectively identified in patient derived samples by Al-Hajj et al. using antibodies against ESA, CD24, and CD44 [8], and later validated for established breast cancer cell lines [14]. The labeling of CICs using antibodies or enzyme substrates [15] has been an invaluable tool to study the features of CICs. Nevertheless, both techniques have disadvantages, which make it difficult to study living CICs *in-situ *or *in-vivo*. To overcome this problem, we recently developed an imaging system for breast and glioma CICs that allows for identification and tracking of CICs without the need for antibodies or exogenous enzyme substrates [16]. It is based on the observation that CICs lack 26S proteasome function. In cell lines engineered to express a fusion protein between the fluorescent protein ZsGreen and the C-terminal degron of murine ornithine decarboxylase (cODC), CICs can be identified via fluorescent imaging due to the accumulation of ZsGreen-cODC protein while non-tumorigenic cells degrade this protein immediately after translation.

In the present study, we hypothesized that CICs, in contrast with their non-tumorigenic counterparts, not only survive fractions of ionizing radiation but also retain the CIC phenotype as defined by operational means and may even become more aggressive than populations of non-irradiated control CICs. Using two distinct marker systems to identify breast CICs (CD24^-/low^/CD44^high^, or lack of proteasome activity) and sphere forming assays, developed by Dontu et al., to enrich for normal mammary stem cells/progenitors [17], we demonstrated that cycling cell populations were enriched for CICs after irradiation with fractions of 2 or 3 Gray (Gy). Irradiated CICs not only survived but also retained their self-renewal capacity for at least four generations. CICs were resistant to radiation-induced apoptosis and were arrested in the G2 phase of the cell cycle, while non-tumorigenic cells were prone to radiation-induced apoptosis. Finally, in lineage-tracking experiments we observed that fractionated radiation not only spared CICs but also mobilized CICs from a quiescent/G0 phase of the cell cycle into actively cycling cells, while the surviving non-tumorigenic cells were driven into senescence.

## Materials and methods

### Cell culture

Human MCF-7 and T47D breast cancer cell lines were purchased from American Type Culture Collection (Manassas, VA, USA). MCF-7-ZsGreen-cODC and T47D-ZsGreen-cODC were obtained as described in Vlashi et al. [13]. All cells were cultured in log-growth phase in Dulbecco's Modified Eagle Medium (DMEM) (Invitrogen, Carlsbad, CA, USA) (supplemented with 10% fetal bovine serum (Sigma Aldrich, St Louis, MO, USA) and penicillin and streptomycin cocktail (Sigma Aldrich)) and were grown in a humidified incubator at 37°C with 5% CO_2_.

### Radiation

Cells were irradiated at room temperature with a ^137^Cs laboratory irradiator (Mark I, JL Shephard, San Fernando, CA, USA) at a dose rate of 4.95 Gy/minute for the time required to apply a prescribed dose. For fractionated radiation of 2 or 3 Gy, cells were irradiated on eight or five consecutive days, respectively. The single dose was applied at the same time as the last dose of fractionated radiation. Corresponding controls were sham irradiated. Cell proliferation, number of stem cells, and sphere forming assays were performed 48 h after the last fraction of radiation.

### Flow cytometry

After fractionated radiation, CD24 and CD44 expression was analyzed in cells derived from monolayer cultures following incubation with trypsin-EDTA and passage through a 40 μm sieve. At least 10^5 ^cells were pelleted by centrifugation at 500 × g for five minutes at 4°C, resuspended in 10 μL of monoclonal mouse anti-human CD24-fluorescein isothiocyanate (FITC) antibody (BD Pharmingen, San Jose, CA, USA) and a monoclonal mouse anti-human CD44-phytoerythrin (PE) antibody (BD Pharmingen), and incubated for 20 minutes at 4°C. Corresponding isotype control APC-conjugated antibodies (BD Pharmingen) and isotype control PE-conjugated antibodies (BD Pharmingen) were used as controls (Additional file 1). Three independent experiments were performed.

We had previously shown [13] that breast cancer stem cells could be identified via their low proteasome activity, which can be assessed by analyzing ZsGreen-cODC protein accumulation. For 48 h after the last fraction of radiation, cells were trypsinized and ZsGreen-cODC expression was assessed by flow cytometry. Cells were defined as *ZsGreen-cODC positive *if the fluorescence in the FL-1H channel exceeded the fluorescence level of 99.9% of the empty vector-transfected control cells (Additional file 2).

### Immunofluorescence

Six day-old MCF-7-ZsGreen-cODC mammospheres were transferred on glass slides by centrifugation in a cytocentrifuge (Cytospin, Shandon Elliot, London, UK) and fixed with 4% formaldehyde. The cells were incubated in blocking buffer (2% Bovine Serum Albumin (BSA), in phosphate buffer saline (PBS)) and antibodies (anti-CD44 APC-conjugated antibody (BD Pharmingen) and anti-CD24 PE-conjugated antibody (BD Pharmingen)) were added for one hour at room temperature. Hoechst 33342 (5 μg/mL, Invitrogen) solution was added for nuclear staining. The slides were visualized with an Olympus IX71 inverted fluorescent microscope. Grayscale images were merged using the ImageJ software [18] and displayed in false colors.

### Sphere forming capacity

After irradiation, cells were trypsinized and plated in selection media (pheno-red free DMEM-F12, 0.4% BSA (Sigma), 10 ml of B27 supplement (Invitrogen)/500 ml of media, 5 μg/ml bovine insulin (Sigma), 4 μg/ml heparin (Sigma), 20 ng/ml fibroblast growth factor 2 (bFGF, Sigma) and 20 ng/ml epidermal growth factor (EGF, Sigma)) into 96-well plates, ranging from 1 to 256 cells/well. Growth factors, EGF, bFGF and heparin, were added every three days, and the cells were allowed to form spheres for 20 days. The number of spheres formed per well was then counted and expressed as a percentage of the initial number of cells plated. Cells were also plated in selection media into 100 mm suspension dishes at 10,000 cells/ml, and allowed to form spheres for 15 days, these cells were used for secondary sphere forming experiments. The same protocol was used after the secondary sphere forming assays, in order to plate cells for analyzing the formation of tertiary and quaternary spheres. Three independent experiments were performed.

### Determining the number of cell divisions

We used the red fluorescent lipophilic dye PKH26 for proliferation tracking. Cells were trypsinized to obtain a single cell suspension and stained with the membrane-labeling dye PKH26 according to manufacturer's protocol (Sigma Aldrich). Briefly, cells were washed twice with PBS to remove exogenous proteins. Immediately before staining, cells were dispersed in 1 ml of Diluent C (an iso-osmotic, salt-free staining vehicle) at a concentration twice the desired final staining concentration (20 × 10^6 ^cells/mL). In parallel, a 2× solution of the PKH26 dye was prepared by diluting 4 μl of the ethanolic dye stock solution (4 μM) into 1 ml of Diluent C in a separate tube. Rapidly dispensing and admixing an equal volume of 2× cell solution into the 2× dye solution initiated the staining. After two to three minutes at room temperature, the staining was stopped by adding an equal volume of fetal bovine serum (FBS). Stained cells were then washed twice in medium, counted, and plated at 200,000 cells per 100 mm culture dish. Homogeneous staining between CICs and non-tumorigenic cells was immediately verified by flow cytometry (FL-2 Channel) (Additional file 3), and the mean FL-2 fluorescence at this time was considered as time 0 (Mean FL-2(t_0_)). Forty-eight hours after the last fraction of radiation, cells were trypsinized, and the remaining PKH26 staining was analyzed by flow cytometry [Mean FL-2(t_sample_)]. The number of cell divisions was calculated using the following formula:

### Cell cycle and G0 phase analysis

After fractionated radiation, cells were stained according to Darzynkiewicz et al. [19]. Briefly, cells were trypsinized and rinsed with HBSS/1 mM HEPES/10% FBS. DNA was stained with 1 μg/ml Hoechst 33342 in HBSS/1 mM HEPES/10% FBS solution/50 μM Verapamil, 45 minutes at 37°C, followed by RNA staining with 3.3 μM pyronin Y (PY) for 45 minutes at 37°C. Finally, cells were rinsed with HBSS/1 mM HEPES/10% FBS solution, and resuspended in PBS. At least 100,000 cells were analyzed by flow cytometry. ZsGreen-cODC expression was analyzed in FL-1H, PY in FL-2H and Hoechst 33342 in FL-5A.

### β-galactosidase staining for cellular senescence

β-galactosidase-positive cells were detected by the method of Dimri et al. [20]. Briefly, cells were washed two times with PBS and then fixed with PBS/2% formaldehyde/0.2% glutaraldehyde solution for five minutes. Cells were then washed again two times with PBS. After the last wash, staining solution was added (1 mg/ml 5-bromo-4-chloro-3-inolyl-β-D-galactoside (X-gal) (20 mg/ml stock, in dimethyformamide), 40 mM citric acid/sodium phosphate, pH 6.0, 5 mM potassium ferrocyanide, 5 mM potassium ferricyanide, 150 mM NaCl, 2 mM MgCl_2_) and cells were incubated in a 37°C for 18 h. After incubation, cells were washed two times with PBS and visualized with an Olympus IX71 inverted fluorescent microscope. The percentage of β-galactosidase-positive cells was counted in 10 random fields at 20× magnification.

### Statistical methods

All data are represented as means +/- standard error means (SEMs). A *P*-value of ≤ 0.05 in a paired two-sided Student's *t*-test was considered to indicate statistically significant differences.

## Results

### CICs population in breast cancer retain self-renewal capacity and multi-lineage potency after fractionated radiation

**Figure 1:**
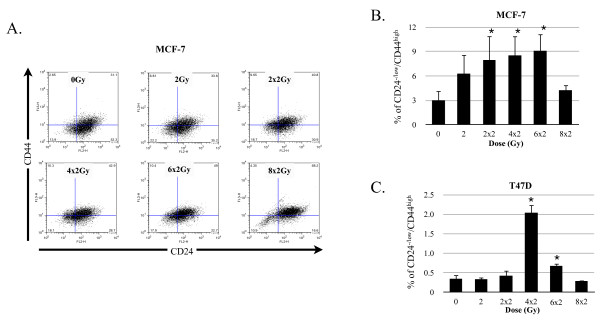
**Radiation response of cells in adherent monolayer cultures**. Fluorescence-activated cell-sorting (FACS) analysis to measure CD44 and CD24 expression of cells derived from MCF-7 monolayer cultures. (**A**) FACS dot plots are shown from one representative experiment in MCF-7. Bar graphs show the means (+/- SEM) from three independent experiments with MCF-7 (**B**) and T47D (**C**).

We have previously reported that fractionated radiation (5 × 3 Gy) increased the number of breast CICs in established cell lines by approximately two-fold. However, this increase was restricted to cells floating on top of monolayer cultures and it was not shown for the adherent cell population [9]. In the present study, we looked into possible increases in CSC numbers in the adherent population, focusing on the kinetics of changes in the size of the CICs population. Monolayer cultures of MFC-7 and T47D breast cancer cells were grown under standard conditions and irradiated with 0, 2, 2 × 2, 4 × 2, 6 × 2, or 8 × 2 Gy in daily fractions. After the last fraction, the cells were incubated for another 48 hours to simulate a typical weekend treatment gap applied in clinical radiation oncology. The size of the CD24^-/low^/CD44^high ^population was analyzed by flow cytometry (Figure 1A, Table 1). For MCF-7 cells (Figure 1A, B, Table 1) the largest increase in the percentage of CD24^-/low^/CD44^high ^cells was seen 48 hours after the 6 × 2 Gy treatment was applied (three-fold increase, *P *= 0.029). However, the CD24^-/low^/CD44^high ^cell numbers subsequently returned to control levels after another two fractions of 2 Gy were applied, indicating that at a dose of 8 × 2 Gy eliminates CICs population and non-tumorigenic cells at the same rate. Interestingly, we also observed an increase in the percentage of CD24^-/low^/CD44^high ^cells for the T47D cell lines (Figure 1C, Table 1) however, with slightly different kinetics. In T47D, the maximum increase of CD24^-/low^/CD44^high ^cell population was observed 48 hours after a fractionated dose of 4 × 2 Gy was applied (6.9-fold increase, *P *= 0.001). Similarly to MCF-7, eight daily fractions of 2 Gy seemed to eliminate CICs and non-tumorigenic cells at the same rate.

**Table 1:** Comparison of fractionated irradiation effect on CIC population, CICs markers and corresponding sphere forming capacity.

MCF-7					
CD24^Low/-/^CD44^Hight^			ZsGreen-cODC^+^		
**Dose**	**% of CICs (+/- SEM)**	**Sphere forming capacity**	**Sphere forming capacity**	**% of CICs (+/- SEM)**	**Dose**

0	3.01 (+/- 0.61), *P *value	9.4 (+/- 0.5), *P *value	12.32 (+/- 2.01), *P *value	2.17 (+/- 0.47), *P *value	0

2	6.54 (+/- 1.95), *P *= 0.095	13.49 (+/- 1.32), *P *= 0.01	15.25 (+/- 3.2), *P *= 0.661	3.70 (+/- 0.52), *P *= 0.051	3

2 × 2	8.04 (+/- 1.47), *P *= 0.036	10.76 (+/- 0.96), *P *= 0.137	17.54 (+/- 4.15), *P *= 0.096	3.13 (+/- 0.53), *P *= 0.252	2 × 3

4 × 2	8.56 (+/- 1.21), *P *= 0.020	11.85 (+/- 1.81), *P *= 0.198	17.93 (+/- 2.33), *P *= 0.051	6.73 (+/- 1.33), *P *= 0.141	3 × 3

6 × 2	9.10 (+/- 1.04), *P *= 0.029	11.85 (+/- 1.48), *P *= 0.035	24.52 (+/- 4.50), *P *= 0.048	11.26 (+/- 2.06), *P *= 0.091	4 × 3

8 × 2	4.78 (+/- 0.34) *P *= 0.229	11.85 (+/- 1.27), *P *= 0.002	25.37 (+/- 5.2), *P *= 0.039	12.64 (+/- 1.91), *P *= 0.044	5 × 3

					

**T47D**					

**CD24^Low/-^/CD44Hight**			**ZsGreen-cODC+**		

**Dose**	**% of CICs (+/- SEM)**	**Sphere forming capacity**	**Sphere forming capcity**	**% of CICs (+/- SEM)**	**Dose**

0	0.35 (+/- 0.06), *P *value	9.46 (+/- 2.20), *P *value	3.35 (+/- 2.01), *P *value	2.47 (+/- 0.88), *P *value	0

2	0.34 (+/- 0.03), *P *= 0.868	6.74 (+/- 1.31), *P *= 0.014	3.13 (+/- 2.69), *P *= 0.231	4.31 (+/- 1.13), *P *= 0.029	3

2 × 2	0.43 (+/- 0.08), *P *= 0.471	13.76 (+/- 4.01), *P *= 0.146	6.48 (+/- 3.14), *P *= 0.091	9.72 (+/- 2.81), *P *= 0.068	2 × 3

4 × 2	2.04 (+/- 0.12), *P *= 0.001	12.74 (+/- 4.58), *P *= 0.157	9.96 (+/- 2.33), *P *= 0.049	16.09 (+/- 5.57), *P *= 0.103	3 × 3

6 × 2	0.68 (+/- 0.04), *P *= 0.007	17.99 (+/- 5.17), *P *= 0.027	12 (+/- 2.66), *P *= 0.033	24.06 (+/- 4.15), *P *= 0.025	4 × 3

8 × 2	0.29 (+/- 0.01), *P *= 0.449	18.92 (+/- 5.83), *P *= 0.018	7.65 (+/- 2.55), *P *= 0.055	21.133 (+/- 1.87), *P *= 0.015	5 × 3

**Figure 2:**
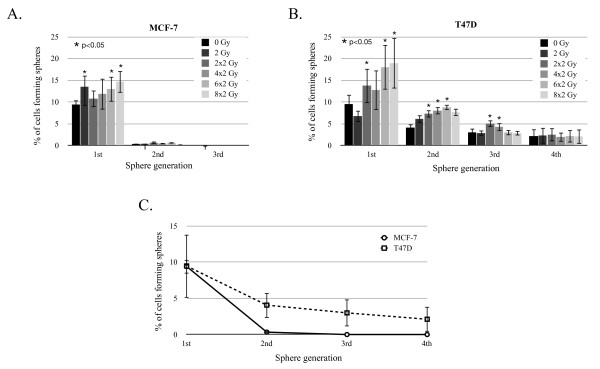
**Long term self-renewal capacity after fractionated radiation**. CICs number was evaluated by sphere forming capacity for MCF-7 (**A**) and T47D (**B**) for four sequential generations. (**C**) Maintenance of self-renewal capacity of untreated breast CICs over four generations. Means (+/- SEM) of at least three independent experiments are shown.

The generation of mammospheres from single cells seeded at clonal densities has been proven a valuable tool for assessing the self-renewal capacity of CICs [14]. Therefore, we employed this strategy to analyze the effect of radiation on the self-renewal capacity of breast cancer stem cells in two different cell lines, MCF-7 and T47D. Both cell lines were irradiated with a single dose, or daily fractions of 2 Gy, and 48 hours after the last dose the irradiated cells and controls were plated into serum-free conditions at clonal densities. After the cells were allowed to form spheres for 20 days, sphere-forming capacity was analyzed by counting the spheres formed. The primary sphere forming capacity of the MCF-7 cell line followed a biphasic pattern, with a significant increase in sphere-forming capacity when the cells were treated with a single dose of 2 Gy (*P *= 0.01), and a fractionated dose of 6 × 2 Gy (*P *= 0.035) or 8 × 2 Gy (*P *= 0.002), while the intermediate doses of 2 × 2 Gy (*P *= 0.14) and 4 × 2 Gy (*P *= 0.20) did not have an effect on sphere-forming capacity (Figure 2A). Secondary sphere formation seemed to also follow the same biphasic pattern, although none of the treatments led to a significant change in sphere-forming capacity. Secondary sphere-formation was only slightly increased in cells derived from primary spheres treated with 2 × 2 Gy (*P *= 0.06).

In contrast to the biphasic pattern of the primary sphere formation in irradiated MCF-7 cells (Figure 2A), we observed a dose-dependent increase in primary sphere formation in T47D cells, with a two-fold (*P *= 0.018) maximum increase at the highest dose of 8 × 2 Gy (Figure 2B). T47D secondary sphere-formation was also dose-dependent. In contrast to MCF-7 cells, which achieved a maximal effect in secondary sphere formation at 2 × 2 and 8 × 2 Gy (1.9- and 1.8-fold increase respectively), sphere formation in T47D was maximal after 6 × 2 Gy (2.2-fold increase, *P *= 0.037) (Figure 2B).

Interestingly, even though the primary sphere-forming capacity was comparable for non-irradiated control cells from both cell lines, we failed to propagate spheres from MCF-7 cells beyond the second generation (Figure 2A, C). However, we were able to generate tertiary and quaternary spheres from the T47D cells. Tertiary sphere formation in this cell line continued to show a significant increase when cells were treated with 2 × 2 or 4 × 2 Gy (*P *= 0.001 and *P *= 0.006 respectively). All other radiation treatments had no effect on tertiary sphere formation (Figure 2B). Finally, in the fourth generation of spheres, radiation-induced increases in the number of CICs were lost and the mean frequency of CICs was 2.16% regardless of the radiation dose applied (Figure 2B).

### Low proteasome activity characterizes a sub-population of CD24^-/low^/CD44^high ^breast CICs

Recently, we demonstrated that glioma and breast cancer initiating cells present a lack of 26S proteasome activity [13]. Therefore, the MCF-7 and T47D cell lines were engineered to express the fusion protein between the green fluorescent protein ZsGreen and the C-terminus of the murine orthinine decarboxylase (cODC). The latter is recognized by the 26S proteasome in an ubiquitin-independent manner, leading to instant degradation of the fusion protein in cells with proteasome activity but accumulates in CICs, which lack proteasome activity.

**Figure 3:**
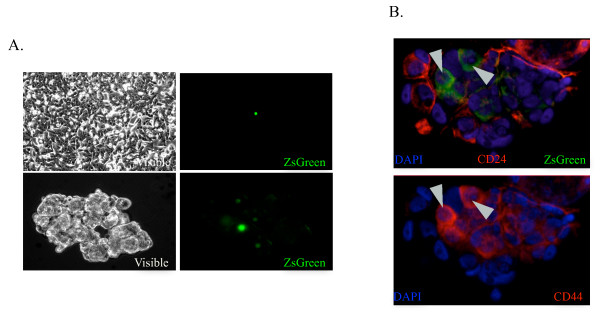
**ZsGreen-cODC^+ ^cells are a subpopulation of CD24^low/-^/CD44^+ ^stem cell-containing population and lead to tumor growth**. (**A**) Confluent MCF-7-ZsGreen-cODC monolayer (top panel) and spheres (bottom panels). ZsGreen-cODC+ cells are enriched in mammospheres. (**B**) Immunofluorescence of CD24 (red, top panel) and CD44 (red, bottom panel) reveals an overlap between ZsGreen-cODC-positive cells and the subpopulation CD24^low/-^/CD44^+ ^in MCF-7-ZsGreen-cODC mammospheres.

As expected, when breast cancer cells were enriched for cancer stem cells by growing them as mammospheres under serum-free conditions, the number of ZsGreen-cODC^+ ^cells increased (Figure 3A). In order to compare the CD24^-/low^/CD44^high ^population with the ZsGreen-cODC^+ ^population, we stained the engineered cells for CD24 and CD44 surface markers. ZsGreen-cODC^+ ^cells did not express CD24 (Figure 3B, top panel), but expressed a high level of CD44 (Figure 3B, bottom panel). However, not all CD24^-/low^/CD44^high ^cells were positive for ZsGreen-cODC, indicating that ZsGreen-cODC^+ ^cells constituted a sub-population of the CD24^-/low^/CD44^high ^population.

In the following studies, we used this intrinsic property of breast CICs to accumulate fluorescent ZsGreen-cODC protein to identify and track breast CICs.

### Fractionated radiation increases the population of CICs with low proteasome activity in breast cancer

In order to confirm the effect of fractionated radiation on CICs observed using CD24^-/low^/CD44^high ^CICs markers, we used the intrinsic low proteasome activity of CICs to re-evaluate the effect of fractionated radiation on CICs in a heterogeneous monolayer population.

MCF-7 and T47D monolayer cultures, stably expressing ZsGreen-cODC protein were treated with daily fractions of 3 Gy for a total dose of 0, 3, 6, 9, 12, or 15 Gy or a single dose of 8.5 Gy, corresponding to the biological equivalent for 5 × 3 Gy, and the percentage of ZsGreen-cODC^+ ^CICs was analyzed 48 hours after the last dose.

**Figure 4:**
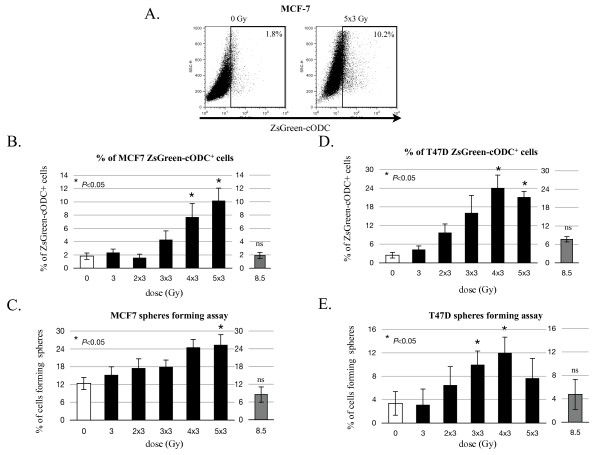
**Radiation response of cells in monolayer cultures using ZsGreen-cODC as a CICs marker**. Fluorescence-activated cell-sorting (FACS) analysis was performed to measure ZsGreen-cODC expression of cells derived from MCF-7 or T47D monolayer cultures after radiation treatment. (**A**) Representative dot blots of control and 5 × 3 Gy treatment of MCF-7 (**a**) or 4 × 3 Gy treatment of T47D (**b**). (**B**) The percent of ZsGreen-cODC-positive (ZsGreen-cODC^+^) cells was estimated by FACS in MCF-7 (**a**) and T47D (**b**) or plated for a sphere forming capacity assay (**C**) for MCF-7 (**a**) and T47D (**b**) cells. White bars: untreated; Black bars: fractionated radiation; Grey bars: biological equivalent dose. Means (+/- SEM) of at least three independent experiments are shown.

Similar to the response to daily fractions of 2 Gy on the percentage of MCF-7 CD24^-/low^/CD44^high ^cells, the lower doses of 3 and 2 × 3 Gy did not affect the number of ZsGreen-cODC^+ ^CICs (*P *= 0.052 for 3 Gy, and *P *= 0.252 for 2 × 3 Gy), while higher doses of 3 × 3, 4 × 3, and 5 × 3 Gy increased ZsGreen-cODC^+ ^CICs up to five-fold in a dose-dependent manner (*P *= 0.044) (Figure 4A, B, Table 1). This was also found in T47D with a ten-fold maximum increase at 4 × 3 Gy (*P *= 0.025) (Figure 4D, Table 1).

To compare the effect of 5 × 3 Gy with a biological equivalent single dose, we also irradiated cells with a single dose of 8.5 Gy at the same time the last fraction of radiation was applied. Interestingly, while a fractionated regimen of 5 × 3 Gy increased the CICs population, the biologically equivalent single dose of 8.5 Gy did not affect this population (MCF-7: *P *= 0.5; T47D: *P *= 0.12) (Figure 4B, D), indicating that tumorigenic (CICs) and non-tumorigenic cells have the same sensitivity to single high doses of radiation.

Next, in order to compare the effect of daily 2 Gy and 3 Gy fractions treatment on CICs population, we performed sphere-forming capacity assays with cells irradiated with 3 Gy daily fractions or a single dose of 8.5 Gy. Forty-eight hours after the last dose of radiation, cells were trypsinized and plated at clonal densities in serum-free conditions. After three weeks, mammospheres were counted to evaluate the effect of 3 Gy daily fractions on sphere formation/number of CICs. Again, 4 × 3 or 5 × 3 Gy induced a maximum increase in MCF-7 sphere formation by 3.6-fold (*P *= 0.033) and a two-fold increase in T47D (*P *= 0.039) (Figure 4C, E, Table 1). In contrary, a single dose of 8.5 Gy did not affect sphere formation in MCF-7 (*P *= 0.134) or in T47D (*P *= 0.098) (Figure 4C, E).

### Fractionated radiation induced proliferation of a subpopulation of CICs

**Figure 5:**
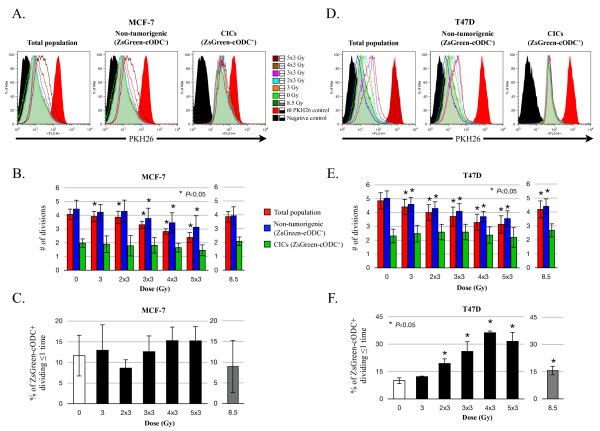
**Effect of fractionated radiation on the proliferation of CICs**. ZsGreen-cODC expression and cell membrane PKH26 staining was analyzed in MCF-7 or T47D monolayer cultures before and after radiation treatment. Cells were stained with PKH26, plated, and treated with the appropriate radiation dose. Representative histograms of PKH26 fluorescence in the total population, non-tumorigenic and CICs populations in MCF-7 (**A**) and T47D (**D**). Calculated numbers of cell divisions in MCF-7 (**B**) and T47D (**E**). Red bars: Total population; Blue bars: Non-tumorigenic cells; Green bars: CICs. Quantification of non-dividing CICs after fractionated radiation MCF-7 (**C**) and T47D (**F**). White bars: untreated; Black bars: fractionated radiation; Grey bars: biological equivalent 8.5 Gy treatment. Data from means (+/- SEM) of at least three independent experiments are shown.

The increase in the ZsGreen-cODC^+ ^population of CICs could result from absolute or relative increases of cell numbers in this population. In an effort to better understand the nature of this increase, we decided to track the proliferation of ZsGreen-cODC^+ ^CICs and non-tumorigenic ZsGreen-cODC^- ^cells. For this purpose, we labeled the cell membrane using PKH26, and evaluated the mean fluorescence intensity at the time of staining and 48 h after the last dose of radiation by flow cytometry. PKH26 stains the membranes of CICs or non-tumorigenic cells with the same bright homogeneous red fluorescence (Additional file 3). This homogeneous red fluorescence is partitioned between daughter cells during each cell division. Because daughter cell fluorescence intensities are approximately halved after each division, the fluorescent intensity of a cell at a certain time point relative to the fluorescent intensity at the time of initial staining provides information about how many divisions the cell has undergone (Figure 5A, D).

In untreated cells (0 Gy), PKH26 mean fluorescence analysis of the total population indicated that MCF-7 and T47D underwent 4.3 (+/- 0.46) and 4.85 (+/- 0.58) divisions respectively (Figure 5B, E, red bars at 0 Gy) in a seven-days time period, which compares well to the published doubling times of these cell lines [16]. Non-tumorigenic ZsGreen-cODC^- ^cells on average divided 4.88 (+/- 0.58) and 5.03 (+/- 0.54) times in seven days, in MCF-7 and T47D respectively (Figure 5B, E, blue bars at 0 Gy), while the population of CICs ZsGreen-cODC^+ ^showed a much lower number of divisions in this time period with a mean of 2.16 (+/- 0.45) and 2.3 (+/- 0.5) divisions in MCF-7 and T47D respectively (Figure 5B, E, green bars at 0 Gy), during the seven days of the experiment, which was consistent with the slow-cycling nature of CICs [21].

Treatment with fractionated radiation resulted in a dose-dependent increase in the mean fluorescence (Figure 5A, D) indicating declining numbers of cell divisions, from 4.3 (+/- 0.46) and 4.85 (+/- 0.58) divisions at 0 Gy to 2.22 (+/- 0.44, *P *= 0.012) and 3.14 (+/- 0.62, *P *= 0.009) divisions at 5 × 3 Gy in MCF-7 and T47D, respectively (Figure 5B, E). While the decrease in the number of divisions of non-tumorigenic ZsGreen-cODC^- ^cells mostly reflected the decrease in proliferation of the total population of cells (MCF-7: *P *= 0.028; T47D: *P *= 0.008) (Figure 5B, E, red and blue bars), the proliferation of CICs was not significantly affected by fractionated irradiation, with 1.4 (+/- 0.34, *P *= 0.052) divisions occurring in MCF-7 cells, and 2.2 (+/- 0.71, *P *= 0.67) divisions in T47D (Figure 5B, E, green bars).

To further understand how the CICs population could increase after radiation, while their proliferation rate remained the same, we analyzed the radiation response of non-dividing CICs (less than or equal to one division) by evaluating the number of ZsGreen-cODC^+ ^cells which did not lose the PKH26 staining after radiation by flow cytometry (Figure 5C, F). While the non-dividing MCF-7 CICs population remained unchanged with a non-significant increase from 11.64% to 15.23% (*P *= 0.11) (Figure 5C), the non-dividing T47D CICs population increased from 9.99% to a maximum of 36.23% (*P *= 0.003) in a dose-dependent manner (Figure 5F), thus indicating a differential radiation responses of CICs of different origin.

### Fractionated radiation eliminates non-tumorigenic cells and mobilizes CICs from G0 phase into the cell cycle

**Figure 6:**
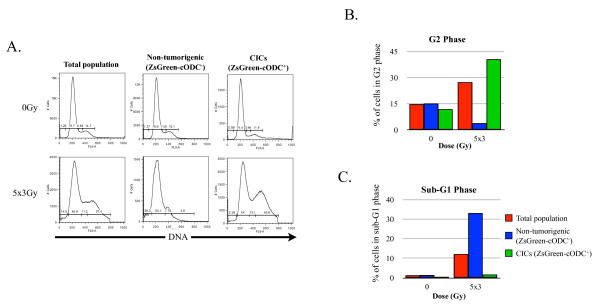
**The effect of fractionated irradiation on cell cycle of non-tumorigenic cells and CICs**. Cell cycle distribution of the total population, non-tumorigenic cells and CICs, 48 h after the last dose of radiation analyzed by FACS. **(A) **Histograms of representative cell cycle analysis, **(B) **G2 phase after 0 and 5 × 3 Gy of fractionated radiation, **(C) **SubG1 fraction after 0 and 5 × 3 Gy of fractionated radiation.

To analyze how radiation increased the number of T47D CICs while a substantial proportion of CICs (36.23%) were not dividing more than one time during the observation period, we analyzed the effect of fractionated radiation on cell cycle distribution and the population of cells in the G0 phase of the cell cycle. Cell cycle analysis revealed induction of a G2 arrest in the total population of T47D cells after 5 × 3 Gy, from 14.7% to 21.5% (Figure 6A, left panels, 6B, red bars). This arrest was not found in the non-tumorigenic cell population, which instead underwent apoptosis (subG1 population increased 24-fold, from 1.37% at 0 Gy to 33.1% at 5 × 3 Gy (Figure 6A, middle panels, 6C blue bars)). Induction of apoptosis in CICs was only minimally increased from 0.59% to 1.67% (Figure 6A, right panels, 6C, green bars). CICs were mostly arrested in the G2 phase of the cell cycle, with an increase of cells in G2 phase from 11.9% to 27.5% (Figure 6A, right panels, 6B, green bars).

Cells in the G0 phase can be distinguished from cells in the G1 phase of the cell cycle based on their RNA content. Cells in G0 phase have the same quantity of DNA as cells in G1 phase but lower amounts of RNA, indicating lower transcriptional cell activity. In order to assess the number of cells in G0, cells were stained with a specific dye for DNA, Hoechst 33342, and a specific dye for RNA (pyronin Y), 48 h after the last dose of radiation and analyzed by flow cytometry.

**Figure 7:**
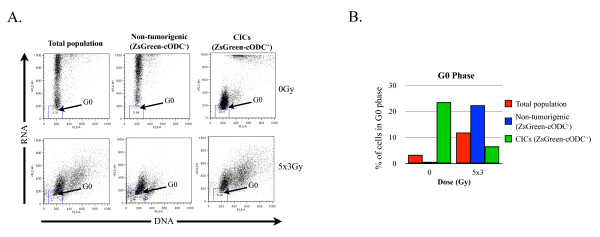
**The effect of fractionated irradiation on G0 phase of non-tumorigenic cells and CICs**. FACS analysis of G0 phase in the total population, non-tumorigenic cells and CICs, 48 h after the last dose of irradiation. Cell DNA was stained with Hoechst 33342, and the RNA was stained with pyronin Y (PY), and cell fluorescence was analyzed on a flow cytometer. (**A**) Dot blots of DNA and RNA content. (**B**) Cells in G0 after 0 and 5 × 3 Gy fractionated irradiation. Data show one representative experiment.

After fractionated radiation with 5 × 3 Gy, the total population of T47D cells showed a 3.6-fold increase of cells in the G0 phase of the cell cycle, from 3.31% to 11.9% (Figure 7A, left panels, 7B, red bars). While the increase of non-tumorigenic cells in G0 phase mostly reflected the increase of G0 phase cells of the total population, from 0.59% at 0 Gy to 22.4% at 5 × 3 Gy (Figure 7A, middle panels, 7B, blue bars), CICs presented the opposite response. In the non-irradiated control sample, approximately 25% of CICs were in G0 phase (Figure 7A, right panels, 7B, green bars), which was consistent with the slow-cycling/quiescent nature of CICs population. After irradiation with 5 × 3 Gy the number of CICs in the G0 phase decreased four-fold to 6.56% (Figure 7A, bottom right panel, 7B, green bars), indicating radiation-induced mobilization of CICs from a G0/quiescent phase into a proliferating state.

### Fractionated radiation induces senescence of non-tumorigenic cells but not of CICs

Cells in G0 phase can be in a quiescent or senescent state. While CICs left the G0 state after radiation to enter the cell cycle, the proportion of surviving non-tumorigenic cells in the G0 phase of the cell cycle increased after radiation. In order to distinguish between senescence of non-tumorigenic cells and quiescence, we performed a specific senescence staining via a β-galactosidase activity assay [20]. Therefore, cells were stained for β-galactosidase activity using the X-Gal substrate 48 h after the last dose of fractionated radiation.

**Figure 8:**
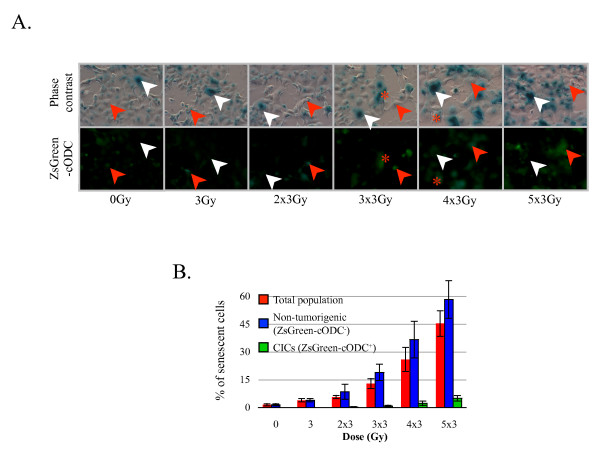
**The effect of fractionated irradiation on senescence induction of non-tumorigenic cells and CICs**. After radiation treatment cells were fixed and stained for β-galactosidase activity. (**A**) Representative pictures of cells after fractionated radiation regimen and β-gal assay. White arrow: quiescent non-tumorigenic cells; red arrow: non-quiescent CICs; red star: quiescent CICs. (**B**) Bar graphs show quantification of senescent cells in T47D. Red bars: total population; blue bars: non-tumorigenic cells; green bars: CICs. Means (+/- SEM) of at least two independent experiments are shown.

At 0 Gy, 2% of the total population was X-gal-positive/senescent cells (Figure 8A, B, red bars). The X-gal-positive (X-Gal^+^) cells were exclusively non-tumorigenic, ZsGreen-cODC^- ^cells, indicating that untreated ZsGreen-cODC^+ ^CICs in G0 phase were indeed quiescent cells and not senescent. Fractionated radiation induced a dose dependent increase of X-Gal^+^/senescent cells in the total population, with a maximal effect at 5 × 3 Gy (25-fold increase, *P *= 0.09) (Figure 8A, B, red bars). While 58% of non-tumorigenic, ZsGreen-cODC^-^, cells were X-Gal^+ ^after 5 × 3 Gy (*P *= 0.106) (Figure 8B, blue bars), senescence was induced in only a small number of ZsGreen-cODC^+ ^CICs (6.4%) only after the maximum radiation dose of 5 × 3 Gy was applied (Figure 8B, green bars). These results suggested that fractionated radiation induces senescence preferentially in non-tumorigenic cells, while CICs are resistant to radiation-induced senescence.

## Discussion

In this study we used two different, and independent, CICs markers (CD24^-/low^/CD44^high ^and lack of proteasome activity) to confirm our previous observation [9] that breast cancer cells are highly enriched for CICs after fractionated radiation. We demonstrated an increase in sphere forming capacity, an operational measure of the number of CICs [14], in cells derived from irradiated breast cancer cultures from two different cell lines, MCF-7 and T47D (Figures 1 and 2; Figure 4B, C, E, F). Since introduced by Dontu et al. [17], sphere forming capacity assays have been widely used to assess self-renewal capacity of cancer stem cells. In theory, a growing sphere derives from a single stem cell. Nevertheless, initially spheres may also derive in part from early progenitors and clumping, thereby introducing errors in the determination of the absolute number of CICs. In order to make our results more accurate, we performed subsequent generations of sphere forming assays. Importantly, in T47D cells the radiation-induced increase in self-renewal capacity was maintained for at least three generations. This ability of breast CICs to retain self-renewal capacity after fractionated radiation was in agreement with our previous studies reporting that fractionated radiation of U87MG xenographs in nude mice increased the Ki-67 labeling index of glioma CICs and increased the ratio of CICs/non-tumorigenic cells in tumors 72 h after the last radiation dose [13]. Our data are also consistent with a previous study demonstrating that glioma CICs were responsible for the re-growth of glioma xenographs after radiation treatment, and that the surviving CD133^+ ^glioma CICs maintained self-renewal capacity [22].

We previously reported that the relative radioresistance of breast CICs resulted from their increased ability to scavenge radiation-induced free radicals, which ultimately cause DNA damage after irradiation [9], an observation reproduced by others independently [10]. While this relative radioresistance can explain relative increases in CICs after radiation, it fails to provide an explanation for absolute increases in CIC numbers. To address this question we focused on the effects of fractionated radiation on proliferation and cell cycle distribution of breast CICs. Here we demonstrate that, consistent with a radiation-induced cell cycle arrest, the mean number of divisions performed by non-tumorigenic breast cancer cells decreased after fractionated radiation, while fractionated radiation did not affect the overall proliferation rate of CICs. Instead, the proliferation rate of the CICs population remained constant regardless of the radiation dose applied (Figure 5B, E). There were two possible explanations for this observation: 1) all the CICs divided at the same rate, and this rate is not affected by fractionated radiation, and 2) one sub-population of CICs stopped to divide after fractionated radiation, while the other sub-population divided faster, resulting in a constant overall proliferation rate of the entire CIC population. Our results indicate that breast CICs of different origin show differential responses to fractionated irradiation with MCF-7 CICs being homogenously radioresistant (Figure 5C), while CICs from the T47D cell line respond heterogeneously (Figure 5F). Furthermore, cell cycle analyses revealed that a substantial number of non-tumorigenic cells were apoptotic after a 5 × 3 Gy (Figure 6) and that a large proportion of the surviving non-tumorigenic cells underwent senescence (Figure 8). In contrast, CICs did not show radiation-induced apoptosis. A sub-population of CICs was arrested in the G2 phase of the cell cycle after fractionated radiation (Figure 6A, B), explaining the increase in the number of non-dividing CICs (Figure 5F). Additionally, the percentage of CICs in the G0 phase significantly decreased after radiation treatment. This indicated that induction of quiescence or senescence was not an explanation for the radiation-induced increase of the non-dividing sub-population of CICs. Furthermore, our data suggest that after radiation treatment a sub-population of CICs, which were initially quiescent (G0), were recruited into the cell cycle. Sham and Durand have previously demonstrated that fractionated irradiation of spheroid cells (now recognized to be enriched for CICs) presented an un-modified growth fraction but a dramatic decrease of cell loss factor, 48 h after the last dose [23]. Considering our observations this decrease of cell loss factor, at the 48 h time point, could be explained by the enrichment for and the dramatic increase in proliferation of the CIC population, which could mask the loss of non-tumorigenic cells caused by induction of apoptosis.

## Conclusions

While we and others previously described the radiation resistance phenotype of CICs, the extent of the remaining self-renewal capacity of the CIC population after fractions of radiation was unknown. In this study, we demonstrated that radiation resistant CICs in breast cancer are not only viable after treatment with fractionated radiation, but retain their self-renewal capacity over several generations. Most importantly, the increase of their self-renewal capacity indicated that irradiated CICs may become more aggressive than populations of non-irradiated CICs. Therefore, since the breast CIC population contains a population of cells with the capacity to re-populate a tumor, which could be more tumorigenic and metastatic after treatment, it would be very important that this population of breast cancer cells be targeted during fractionated radiation. Such a strategy may offer a novel approach to improve patient outcome in the future.

## Abbreviations

β-gal: β-galactosidase; BSA: Bovine Serum Albumin; CICs: cancer initiating cells; ^137^Cs: Cesium 137; DMEM: Dulbecco's Modified Eagle Medium; EGF: epidermal growth factor; FACS: fluorescence-activated cell-sorting; FBS: fetal bovine serum; bFGF: fibroblast growth factor 2; FITC: fluorescein isothiocyanate; Gy: Gray; HBSS: Hank's buffered salt solution; PBS: Phosphate Buffer Saline; PE: phycoerythrin; SEMs: standard error means; ZsGreen-cODC: fused ZsGreen fluorescent protein and C-terminal degron of ornithine decarboxylase.

## Competing interests

The authors declare that they have no competing interests.

## Authors' contributions

CL participated in the design of the study, carried out the cell cultures, radiation experiments, FACS analysis and senescence studies, performed the statistical analysis and drafted the manuscript. EV carried out the radiation experiments and participated in the FACS analysis, and the drafting of the manuscript. LDD participated in drafting the manuscript. YHM and CD performed cells culture and sphere-forming capacity assays. KK helped draft the manuscript. FP conceived of the study, designed and coordinated all experiments and helped draft the manuscript. All authors read and approved the final manuscript.

## Supplementary Material

Additional file 1 **Figure S1. Control gate of CD24 and CD44 analysis (Figure **1**)**. Fluorescence-activated cell-sorting (FACS) analysis was performed to measure non-specific binding of anti-mouse isotype control PE-conjugated and anti-mouse isotype control APC-conjugated antibodies, and effects of radiation treatment on cell auto-fluorecence. Click here for file

Additional file 2 **Figure S2. Control gate of ZsGreen-cODC analysis (Figure **4**)**. Cells stably transfected with an empty control vector were irradiated with 5 × 3 Gy (right panel) or sham irradiated (left panel), and cells were analyzed for green auto-fluorescence. Cells were defined as *ZsGreen-cODC positive *if the fluorescence in the FL-1H channel exceeded the fluorescence level of 99.9% of the empty vector control cells. Click here for file

Additional file 3 **Figure S3. Control gate of cell membrane PHK26 staining efficiency (Figure **5**)**. After cell membrane staining with PKH26 fluorescence was analyzed in the total population (left panel), non-tumorigenic cells (middle panel), and CICs (right panel). No difference was found for PKH26 efficiency staining between non-tumorigenic cells and CICs. Click here for file
