# Supplementary figures and images for: Survival and self-renewing capacity of breast cancer initiating cells during fractionated radiation treatment
Source: Breast Cancer Res. 2010 Feb 16;12(1):R13. doi: 10.1186/bcr2479 (PMC2880434; doi:10.1186/bcr2479)

# MCF-7

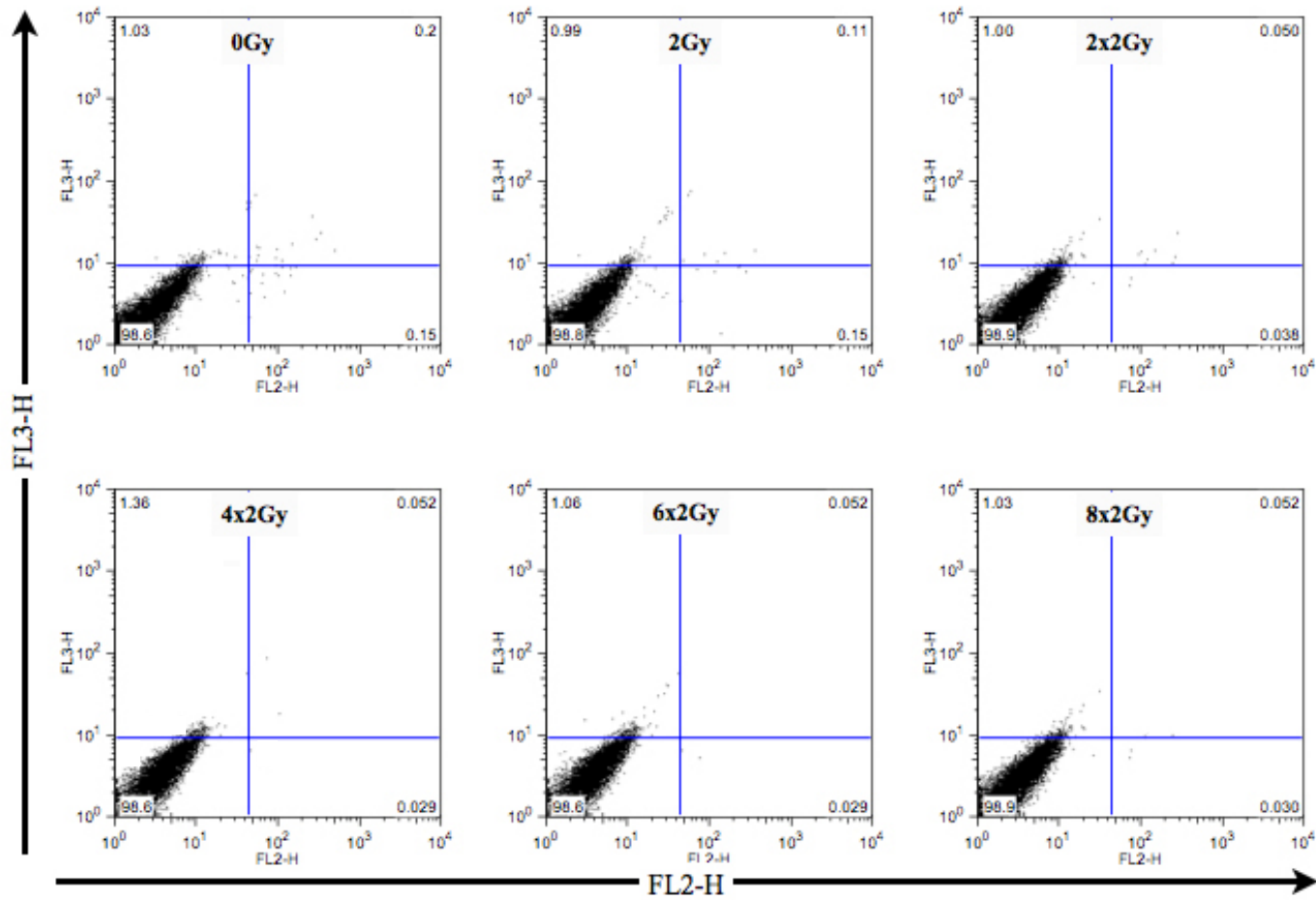

Supplementary Figure 1

Supplement: Additional file 1 — Figure S1. Control gate of CD24 and CD44 analysis (Figure 1). Fluorescence-activated cell-sorting (FACS) analysis was performed to measure non-specific binding of anti-mouse isotype control PE-conjugated and anti-mouse isotype control APC-conjugated antibodies, and effects of radiation treatment on cell auto-fluorecence. [file bcr2479-S1.pdf]

**MCF-7**

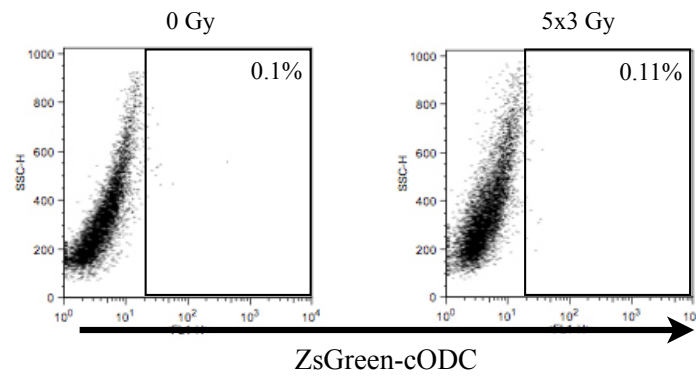

Supplement: Additional file 2 — Figure S2. Control gate of ZsGreen-cODC analysis (Figure 4). Cells stably transfected with an empty control vector were irradiated with 5 × 3 Gy (right panel) or sham irradiated (left panel), and cells were analyzed for green auto-fluorescence. Cells were defined as ZsGreen-cODC positive if the fluorescence in the FL-1H channel exceeded the fluorescence level of 99.9% of the empty vector control cells. [file bcr2479-S2.pdf]

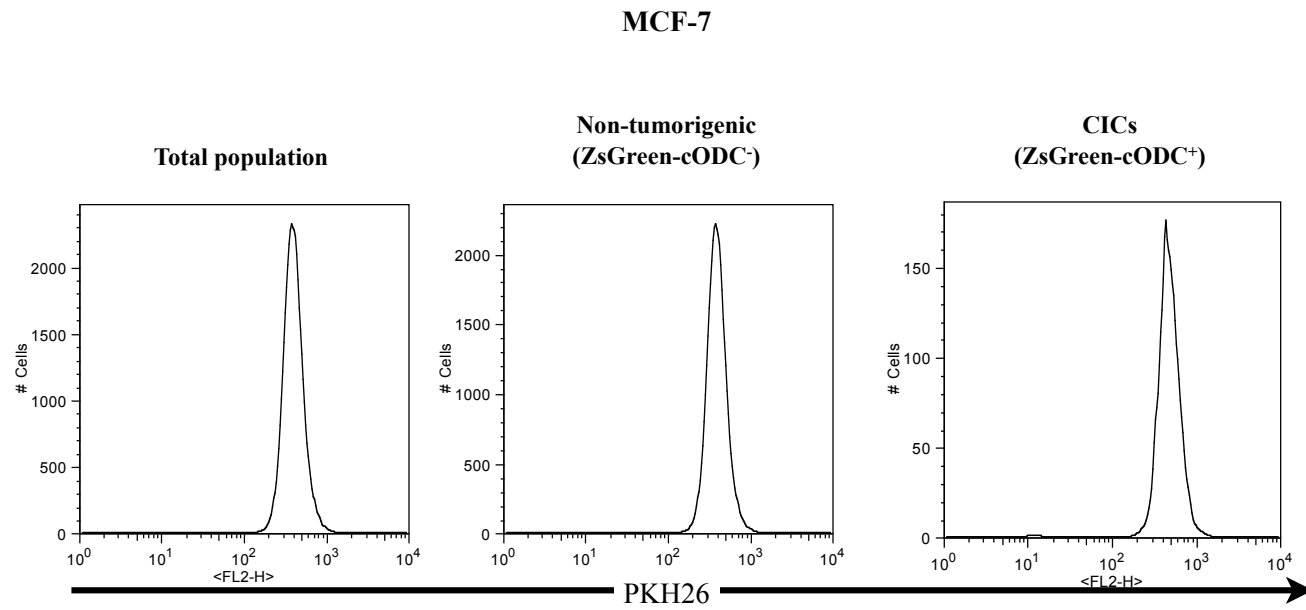

Supplementary Figure 3

Supplement: Additional file 3 — Figure S3. Control gate of cell membrane PHK26 staining efficiency (Figure 5). After cell membrane staining with PKH26 fluorescence was analyzed in the total population (left panel), non-tumorigenic cells (middle panel), and CICs (right panel). No difference was found for PKH26 efficiency staining between non-tumorigenic cells and CICs. [file bcr2479-S3.pdf]
